# Sequence Variation in Multidrug-Resistant Plasmid pLUH01, Isolated from Human Nasopharyngeal Swabs

**DOI:** 10.1128/MRA.00835-18

**Published:** 2018-07-12

**Authors:** Kate V. Atkinson, Lisa A. Bishop, Glenn Rhodes, Nicolas Salez, Neil R. McEwan, Matthew J. Hegarty, Julie Robey, Nicola Harding, Simon Wetherell, Robert M. Lauder, Roger W. Pickup, Mark Wilkinson, Derek Gatherer

**Affiliations:** a Division of Biomedical & Life Sciences, Faculty of Health & Medicine, Lancaster University, Lancaster, United Kingdom; b Royal Lancaster Infirmary, Lancaster, United Kingdom; c Centre for Ecology & Hydrology, Lake Ecosystems Group, Lancaster Environment Centre, Lancaster University, Lancaster, United Kingdom; d UMR_D 190, Emergence des Pathologies Virales, Aix-Marseille University, Marseille, France; e Institute of Biological, Environmental & Rural Sciences, Aberystwyth University, Aberystwyth, United Kingdom; f Queen Square Medical Practice, Lancaster, United Kingdom; Loyola University Chicago

## Abstract

Three variants of the multidrug-resistant plasmid pLUH01 were assembled by deep sequencing from nasopharyngeal swabs. All have a 21-bp deletion in the RS14515 hypothetical gene. Variants 1 through 3 have 2, 6, and 3 nucleotide substitutions, respectively, compared to the pLUH01 reference genome. We named the new plasmid variants pLUH01/Lancaster/2015/1 to pLUH01/Lancaster/2015/3.

## Abstract (precis)

Three variants of the multidrug-resistant plasmid pLUH01 were assembled by deep sequencing from nasopharyngeal swabs. All have a 21-bp deletion in the RS14515 hypothetical gene.

## ANNOUNCEMENT

Lindqvist et al. first sequenced pLUH01 (NCBI reference sequence number NC_017346) as part of a study on multidrug-resistant strains of Staphylococcus aureus (1). pLUH01 is 2,241 bp in length with two genes of known function, *rep*, encoding a replication protein, and *qacC*, encoding a small-molecule efflux transporter conferring resistance to quaternary ammonium compounds. A third open reading frame encodes a hypothetical protein. Plasmids pSK108 (reference sequence number NC_013395) and pKH8 (GenBank accession number U50077) (2) are 0.4% and 0.5% divergent from pLUH01, respectively. More distant relatives include pSA1308 (reference sequence number NC_007928) (2), pWBG754 (reference sequence number NC_013350), pKH4 (GenBank accession number U81980), and pNVH01 (reference sequence number NC_004562). The last two of these have *qacJ* in place of *qacC*, at 27% amino acid divergence (3).

Volunteers were recruited from a general practice surgery and a general hospital in Lancaster, United Kingdom (54.05°N, 2.80°W). Ethical approval was granted by the UK National Research Ethics Service (reference 14/LO/1634, NIHR Clinical Research Network [UKCRN] Portfolio, identification number 17799). All relevant guidelines and regulations were observed. Nasopharyngeal swabs were taken between 16 December 2014 and 25 February 2015. Nucleic acid was extracted from 51 swabs. Sequencing library preparation was performed in March 2015.

Deep sequencing was performed using an Illumina Nextera XT library and HiSeq 2500 system (SRA accession number SRP092324) (4, 5). The variant sequences were assembled from the following sequence pools: pLUH01/Lancaster/2015/1 from 2 pediatric patients with respiratory symptoms (BioSample accession number SAMN05954284) and from 10 asymptomatic adults (SAMN05954287); pLUH01/Lancaster/2015/2 from 6 chronic obstructive pulmonary disease (COPD) patients (SAMN05954289); and pLUH01/Lancaster/2015/3 from a single asymptomatic adult (SAMN05954290). Assembly was performed using Bowtie 1.1.1 (6), using reference sequence number NC_017346 as template (parameters, bowtie –solexa-quals -S -p 8). Average and maximum coverage depths, in reads, for the 3 variants were 127 and 318, 7 and 24, and 24 and 66, respectively. All 3 sequence variants are 2,220 bp long, having a 21-bp deletion starting at position 1933 in comparison to reference sequence number NC_017346. This removes a 21-bp/7-amino-acid tandem repeat in the hypothetical protein. Substitutions are observed at positions (numbers as in reference sequence number NC_017346) 780 (all variants), 1306 (all variants), 1615 (variant 2), 1642 (variant 2), 1663 (variants 2 and 3), and 1678 (variant 2). All of them are synonymous substitutions in the replication protein, except position 780, which is in a noncoding region.

Resistance of Staphylococcus aureus to the quaternary ammonium compound ethidium bromide was first detected in 1969 (7), and plasmids encoding resistance to similar compounds, present in disinfectants and antiseptics typically used in hospitals, have become important subjects of study in the field of antimicrobial resistance (reviewed in reference 8). We assembled full-length pLUH01 variants from 4 of our 9 nasopharyngeal swab sequencing pools (4) and detected fragments in the remaining 5, suggesting widespread prevalence of this plasmid in the population. Although none of our sequence variants has nonsynonymous point substitutions and the significance of the 7-residue deletion in the hypothetical protein remains a matter for speculation, the presence of such variation in samples collected from a single town within a time window of a few months highlights the evolutionary potential of pLUH01 as an antimicrobial resistance determinant.

BAM files are available from https://doi.org/10.17635/lancaster/researchdata/220.

### Data availability.

The complete sequences of pLUH01/Lancaster/2015/1 to pLUH01/Lancaster/2015/3 have been deposited in GenBank under the accession numbers MH251945 to MH251947.
